# Applied Behavior Analysis: Key Points to Improve Autism Treatment

**DOI:** 10.31083/AP38858

**Published:** 2025-03-07

**Authors:** Sergio Machado, Flávia Paes, João Lucas Lima

**Affiliations:** ^1^Center of Neuroscience, Neurodiversity Institute, 26325-010 Queimados, Brazil; ^2^Laboratory of Panic and Respiration, Institute of Psychiatry, Federal University of Rio de Janeiro (IPUB/UFRJ), 22290-140 Rio de Janeiro, Brazil

Although applied behavior analysis (ABA) is recognized as one of the most effective interventions in the treatment of autism spectrum disorder (ASD) [1], its integration with other therapies has proven to be a promising strategy. By combining ABA with speech and occupational therapies, and psychotherapy, it is possible to offer comprehensive and multidisciplinary care, addressing the diverse needs of each autistic individual [2].

Speech therapy, for example, can be integrated with ABA to promote the development of language and social interaction skills [2]. In this context, ABA techniques are used to teach the fundamentals of communication systematically and motivationally, while speech therapy works on specific language aspects, such as articulation, comprehension, and verbal expression. Similarly, occupational therapy can complement ABA, focusing on the development of self-care skills, fine and gross motor coordination, and sensory integration [3]. This integration allows the autistic individual to acquire and generalize these skills in their natural environments, improving their independence and quality of life. Finally, psychotherapy can be incorporated into ABA to work on emotional and behavioral aspects, such as emotion regulation, anxiety reduction, and the promotion of meaningful social interactions [4]. This integration contributes to the autistic’s socio-emotional development, strengthening their ability to cope with the challenges of autism. By integrating these different therapeutic perspectives, the multidisciplinary team can offer a holistic treatment, covering the multiple needs of the child with autism and maximizing their development potential [1].

ABA intervention has increasingly benefited from technological innovations, which have enriched and complemented this approach in the context of autism [5]. From mobile applications and virtual reality systems to the use of artificial intelligence, these tools have demonstrated great potential in various stages of treatment. Mobile applications, for example, can be used to teach specific skills in a playful and interactive way, taking advantage of the strong interest of autistic individuals in digital devices [6]. These applications offer personalized exercises, immediate feedback, and practice opportunities, reinforcing the objectives of ABA intervention. Virtual reality has been explored as a tool capable of simulating realistic environments and situations, allowing the autistic individual to practice and generalize social, communication, and self-care skills in a controlled and safe environment [7]. This technology enables gradual exposure to challenging situations, with the support of the therapeutic team.

Furthermore, artificial intelligence has been used to enhance the collection and analysis of data during the ABA intervention [8]. Automated monitoring systems can identify behavioral patterns, detect progress, and provide valuable information to the therapeutic team, optimizing decision-making and adjusting treatment plans. These technological innovations, when strategically integrated into ABA practice, have the potential to increase the autistic’s engagement, further personalize the intervention, and generate valuable insights for the continuous improvement of the treatment [9].

The active involvement of the family is essential for the success of ABA intervention in the treatment of ASD [1]. This therapeutic approach goes far beyond clinical sessions, requiring the participation and empowerment of all the environments in which the child is inserted. The family plays a crucial role in the therapeutic process. Parents and caregivers are guided and trained to apply ABA techniques in everyday life, ensuring the generalization of the skills acquired by the child in their natural contexts [10]. This close collaboration between professionals and family members allows for a cohesive and consistent approach, maximizing the results of the treatment.

ABA has consolidated itself as an essential approach in the treatment of ASD, promoting significant advances in the development of essential skills and improving the quality of life of autistic individuals [1]. By integrating ABA with other therapies, incorporating technological innovations, and actively involving the family, it is possible to offer a holistic and personalized treatment, maximizing the potential of each autistic individual [1, 2]. This multidimensional approach is crucial to ensure better results and opportunities for social inclusion for ASD children.
